# The Geographical and Climatic Relations of Pneumonia

**Published:** 1882-07

**Authors:** 


### THE GEOGRAPHICAL AND CLIMATIC RELATIONS OF PNEUMONIA.

In the *American Journal of the Medical Sciences* for July, Dr. E. Sanders, of this city, has a valuable statistical paper upon the above subject, to which the following conclusions are appended :

1. The relations of pneumonia to altitude are definite and marked ; with increase in elevation above the level of the sea, there is a steady diminution in the death-rate of pneumonia. To this rule some exceptions exist, but in the large majority of instances the relation holds good.

2. The mean annual rain-fall of a place bears no positive relation to pneumonia ; in some instances a large mortality from the disease coincides with a large precipitation of rain, in others with a small precipitation, while in as many others the contrary conditions are found to prevail.

3. The higher the death-rate of a place from all causes, the greater the mortality from pneumonia. The rule is almost, if not actually, absolute.

4. The larger the actual population of a locality, the greater its relative death-rate from pneumonia ; the explanation for this being probably found in the following : Density of population bears an undoubted relation to the pneumonia-rate, increase in the former being followed by, or going hand-in-hand with, increase in the latter.

5. There is a direct, positive, and unequivocal relation between the mean annual temperature of a place and its death-rate from pneumonia ; the rule being that a high mortality from the disease coincides with a high mean annual temperature. Exceptions exist, but being unusual and rather rare, their existence can hardly be considered to invalidate this rule.

6. Proximity to large bodies of water, such as lakes, inland seas, or the ocean, exerts no appreciable influence on the pneumonia-rate.

7. For North America, pneumonia increases in frequency as we pass from east to west ; for Europe, as we advance from west to east, the rate of increase being very nearly twice as great in the case of the latter as in that of the former.

8. Pneumonia, all other things being equal, increases in frequency the further we advance from the polar regions toward the tropics ; this, however, only up to a certain parallel, beyond which it seems to become less and less commonly met with, until at or near the equator, where it apparently disappears. As far as the latter part of this statement is concerned, such would seem to be the truth, judging by what few facts are at hand. Statistics for the equatorial regions are rare, and even then unreliable ; hence I have purposely omitted to present them. So few, vague and indefinite are they as to be almost valueless, allowing only of problematic deductions.

The foregoing conclusions apply more particularly to the northern hemisphere, all the statistics I have collected referring to places north of the equator ; facts, save in a few instances, relating to places in the southern hemisphere being wanting.

